# GATA1 Promotes Gemcitabine Resistance in Pancreatic Cancer through Antiapoptotic Pathway

**DOI:** 10.1155/2019/9474273

**Published:** 2019-04-10

**Authors:** Zhenyu Chang, Yanan Zhang, Jie Liu, Chengjian Guan, Xinjin Gu, Zelong Yang, Qinong Ye, Lihua Ding, Rong Liu

**Affiliations:** ^1^Department of Hepatobiliary and Pancreatic Surgical Oncology, Medical School of Chinese People's Liberation Army, Beijing 100853, China; ^2^Department of Medical Molecular Biology, Beijing Institute of Biotechnology, Collaborative Innovation Center for Cancer Medicine, Beijing 100850, China

## Abstract

Gemcitabine-based chemotherapy is the first-line treatment for pancreatic cancer. However, chemoresistance is a major obstacle to drug efficacy, leading to poor prognosis. Little progress has been achieved although multiple mechanisms are investigated. Therefore, effective strategies are urgently needed to overcome drug resistance. Here, we demonstrate that the transcription factor GATA binding protein 1 (GATA1) promotes gemcitabine resistance in pancreatic cancer through antiapoptotic pathway. GATA1 is highly expressed in pancreatic ductal adenocarcinoma (PDAC) tissues, and GATA1 status is an independent predictor of prognosis and response to gemcitabine therapy. Further investigation demonstrates GATA1 is involved in both intrinsic and acquired gemcitabine resistance in PDAC cells. Mechanistically, we find that GATA1 upregulates Bcl-XL expression by binding to its promoter and thus induces gemcitabine resistance through enhancing Bcl-XL mediated antiapoptosis in* vitro* and in* vivo*. Moreover, in PDAC patients, Bcl-XL expression is positively correlated with GATA1 level and predicts clinical outcomes and gemcitabine response. Taken together, our results indicate that GATA1 is a novel marker and potential target for pancreatic cancer. Targeting GATA1 combined with Bcl-XL may be a promising strategy to enhance gemcitabine response.

## 1. Introduction

Pancreatic ductal adenocarcinoma (PDAC) is one of the most aggressive tumors with poor prognosis [1]. The 6-month recurrence-free survival (RFS) of PDAC remains below 15%, and the overall survival (OS) rate at 5 years less than 8% [2]. The dismal prognosis is largely attributed to extreme chemoresistant phenotype of the tumor [3]. At present, gemcitabine remains a standard chemotherapeutic agent for advanced pancreatic cancer and postsurgery adjuvant therapy [4]. However, most patients developed resistance within weeks of gemcitabine treatment, and under 25% of patients with PDAC benefited from gemcitabine treatment [5].

The mechanisms of gemcitabine resistance include failure of drug uptake and metabolism, activation of DNA repair pathways, resistance to apoptosis, and change of tumor and stromal microenvironment [6]. Particularly, recent studies demonstrated that signaling pathways modulating proliferation, differentiation, apoptosis, invasion, and angiogenesis, including MAPK, PI3K/Akt, and NF-*κ*B, directly or indirectly regulate gemcitabine sensitivity in PDAC cells [7–10]. However, mechanisms related to gemcitabine resistance are not well elucidated. Further understanding of pathways mediating gemcitabine chemoresistance is crucial for developing improved treatments and prolonging patients' survival in pancreatic cancer.

The major action mode of gemcitabine is to induce apoptosis of cancer cells [11]. The profound deregulation of the apoptotic machinery is one of the central events during development of chemoresistance in cancer cells. Gemcitabine-induced apoptosis involves the mitochondria-mediated signaling pathway, which is mainly modulated by B-cell lymphoma-2 (Bcl-2) family proteins [12]. The antiapoptotic Bcl-2 family proteins include Bcl-2, Bcl-XL, Bcl-w, Mcl-1, and BCL2A1 [13]. Bcl-XL is well known as a major antiapoptotic protein in pancreatic cancer. It is frequently upregulated in chemoresistant cells, counteracting the function of proapoptotic proteins [14]. The knockdown of Bcl-XL significantly sensitized pancreatic cancer cells to gemcitabine-mediated apoptosis [15]. Multiple pathways are involved in gemcitabine resistance through targeting Bcl-XL. For instance, MAPK and HIF-1*α* signaling upregulates Bcl-XL level and thus induces gemcitabine resistance in PDAC cells [16–19]. Activated Akt favors cell survival via the direct regulation of Bcl-XL [20, 21]. However, the regulatory network of Bcl-XL expression is still not well clarified.

GATA1 is the founding member of GATA transcription factor protein family, which contains two highly conserved zinc finger domains, N-terminal finger (NF), and C-terminal finger (CF). GATA1 NF has been reported to bind independently to GAT(C/G) sequences, and GATA1 CF binds with high affinity and specificity to (A/T)GATA(A/G) motifs [22]. GATA1 regulates its target genes through binding to consensus DNA sequence. GATA1 was first found critical for the formation of early eosinophil precursors and for differentiation of committed erythroid precursors and megakaryocytes [23]. Recently, GATA1 was reported to be involved in cell growth, apoptosis, tumorigenesis, and aggressiveness of solid tumors. In breast cancer, GATA1 is overexpressed and promotes survivin expression [24]. Furthermore, the interaction of GATA1 and MMP-2 enhanced glioblastoma invasion and migration [25]. We have previously demonstrated that GATA1 promotes breast cancer growth and metastasis through regulating VEGF expression [26], but the role of GATA1 in PDAC remains unexplored.

In this study, we found that GATA1 was upregulated in PDAC patients and correlated with RFS and OS, specifically in patients treated with gemcitabine. Therefore, we further investigated the function of GATA1 in gemcitabine resistance and confirmed that GATA1 induced intrinsic and acquired gemcitabine resistance through regulating Bcl-XL in* vivo* and in* vitro*. Thus, GATA1 inhibition alone or in combination with Bcl-XL inhibition may be a useful strategy for the treatment of gemcitabine-resistant PDAC patients overexpressing GATA1.

## 2. Methods

### 2.1. Plasmids, Lentiviruses, siRNAs, and Reagents

The eukaryotic expression constructs for GATA1 were generated by cloning PCR-amplified full length sequences into pcDNA3 (Invitrogen). GST-fusion protein encoding vectors were constructed by inserting PCR-amplified sequences into pGEX-KG (Amersham Pharmacia Biotech). The Bcl-XL promoter luciferase reporters were obtained by cloning PCR-amplified promoter fragments into pGL4-basic vector (Promega). The mutated Bcl-XL promoter luciferase reporters were constructed by recombinant PCR. Lentiviral vector of GATA1 was constructed by cloning PCR-amplified full length sequences into pCDH (System Biosciences). The short hairpin RNAs (shRNAs) targeting GATA1 and Bcl-XL cDNA were inserted into PSIH-H1-puro (System Biosciences). The small interfering RNAs sharing the same targets with shRNAs were synthetized from GenePharma (Shanghai, China). The sequences for siRNAs and shRNAs are listed in Table S4a.

Specific antibodies against GATA1 (ab28839) and Bax (ab32503) were purchased from Abcam. Anti-Bcl-XL (#2764), anti-cleaved PARP (#5625), anti-cleaved caspase 3 (#9664), and anti-cleaved caspase 9 (#9505) were from Cell Signaling Technology. Anti-*β*-actin (sc-47778HRP) was from Santa Cruz Biotechnology. Gemcitabine (T0251) was obtained from Targetmol.

### 2.2. Clinical Samples

86 pairs of PDAC tissues and adjacent normal pancreas tissues and 86 separate PDAC tissues were obtained from Chinese PLA General Hospital. In the 86 pairs of cases, 59 of them received gemcitabine treatment, and the other 27 pairs were chemonaive. All patients received radical surgery and were diagnosed by pathologic evidence from January 2011 to May 2017. The mean follow-up time is 23.0 months (1.1-86.6 months). Of 172 cases, 119 received adjuvant gemcitabine-based chemotherapy and 53 did not receive any chemotherapy. For Western blot analysis, another 30 pairs of fresh frozen PDAC and adjacent normal tissues were collected. All samples were obtained with the informed consent of patients and with the approval of the Institutional Review Committees of Chinese PLA General Hospital.

### 2.3. Cell Culture and Transfection

Human embryonic kidney cell line HEK 293T, human normal pancreatic duct epithelial cell line HPDE6c7, and human PDAC cell lines (AsPC-1, BxPC-3, CFPAC-1, PANC-1, and SW1990) were purchased from American Type Culture Collection (ATCC) and cultured in corresponding mediums (Gibco) suggested by ATCC.

Transient transfection of plasmids was conducted using Lipofectamine 2000 Reagent according to the manufacturer's recommendations (Invitrogen). Transient transfections of siRNAs were performed using RNAimax Reagent according to the manufacturer's recommendations (Invitrogen). Stable cell lines were obtained by lentiviral transduction with pCDH or PSIH-H1-puro. Lentiviruses were obtained by cotransfecting recombinant lentivirus vectors and pPACK Packaging Plasmid Mix (System Biosciences) into HEK 293T cells with Megatran reagent (Origene). Viral supernatants were collected and filtered 48 hours after transfection and subsequently added to the culture medium of target cells with 8 *μ*g/ml polybrene (Sigma-Aldrich). The target cells were selected for 30 days with 1 *μ*g/ml puromycin 48 h after infection to generate stably cell lines.

### 2.4. Generation of Gemcitabine-Resistant Cells

PANC-1 and CFPAC-1 cells were cultured in medium containing gradually increasing doses of gemcitabine ranging 1-50 *μ*M and 5-150 *μ*M for PANC-1 and CFPAC-1 cells, respectively. After over 3 months of selection, cells were stably passaged in medium with low concentration of gemcitabine (1 *μ*M and 5 *μ*M respectively) for more than 10 generations. The resistance status was confirmed by CCK8 assay.

### 2.5. Western Blot Analysis

Cells and tissues were lysed in RIPA lysis buffer containing protease inhibitors for 30 min. Tissues were grinded into homogenates before lysis. Equal amounts of protein were separated by 10% or 15% SDS-PAGE and transferred to NC membranes. After blocking for 1 h, the membranes were incubated with indicated antibodies and detected by enhanced chemiluminescence.

### 2.6. Cell Proliferation and Cell Viability Assay

For cell proliferation assay, approximately 3000 cells per well were plated into 96-well plates and incubated overnight at 37°C. Cell numbers was assessed by CCK-8 Kit (Dojindo Laboratories) following the manufacturer's protocols after incubating for 0, 24, 48, 72, or 96 h. The absorbance at 450 nm of each well was examined by a microplate reader. For cell viability assay, approximately 1×10^4^ cells per well were plated into 96-well overnight at 37°C. Then cells were treated with different concentrations of gemcitabine for 48 h before CCK8 assay. IC50 values were generated by an online IC50 calculator (http://www.aatbio.com/tools/ic50-calculator/).

### 2.7. Colony Formation Assay

Approximately 1000 cells per well were seeded into 6-well plates and cultured in regular medium at 37°C. For gemcitabine resistance experiment, cells were treated with gemcitabine (1 *μ*M and 5 *μ*M for CFPAC-1 and PANC-1 respectively) before plating. After 10-14 days, cells were fixed in 4% paraformaldehyde for 10 min and stained with 0.1% crystal violet solution for 20 min. Images were scanned and colony numbers were quantified.

### 2.8. Quantitative Reverse Transcription-PCR (qRT-PCR)

Total RNA was extracted using RNAzol regent (Sigma) following the manufacture's protocol. Equal amount of RNA was reverse-transcribed using SuperScript II Reverse Transcriptase (Invitrogen). Real-time quantitative PCR was performed with SYBR-green premix (Takara) on a CFX96 Real-Time PCR detection system. The relative fold change of target mRNAs was normalized to *β*-actin calculated by 2^−∆∆Ct^ method. Primer sequences used were listed in Table S4b.

### 2.9. Dual-Luciferase Reporter Assay

Cells were seeded into 24-well plates at 40~60% confluency and cotransfected with different luciferase constructs and indicated expression vectors or siRNAs and Renilla luciferase plasmid using Lipofectamine 2000 Reagent. 48 h after transfection, the cells were harvested, lysed and analyzed for luciferase activity with dual-luciferase assay kit (Vigorous) according to the manufacture's protocol. Firefly luciferase activity was normalized to Renilla luciferase activity as control of transfection efficiency. Relative luciferase activity was assayed by the luminometer.

### 2.10. Chromatin Immunoprecipitation (ChIP) Assay

ChIP assay was conducted with the Magna ChIP kit as manual described (Millipore). Briefly, CFPAC-1 cells were cross-linked with freshly prepared 1% formaldehyde. Cell lysis, sonicating, dilution, immunoprecipitation, washing, and elution were subsequently performed following the manufacture's protocol. The purified DNA samples were then amplified by qRT-PCR to determine relative enrichment. Primer sequences used were listed in Table S4c.

### 2.11. Flow Cytometry Assay

Cell apoptosis was analyzed using an Annexin V-APC/PI apoptosis assay kit (KeyGEN BioTECH). After being treated with or without gemcitabine for 48 h, cells were digested, washed, resuspended, and stained with Annexin V-APC and PI according to the manufacture's protocol. The apoptotic cells were detected with a flow cytometer (BD Biosciences).

### 2.12. Animal Experiments

Protocols were approved by the Institutional Animal Care and Use Committee of Beijing Institute of Biotechnology. 1×10^7^ PANC-1 cells stably infected with indicated lentiviruses were subcutaneously injected into the left oxter of 7-week-old BABL/c nude mice. Two weeks after implantation, gemcitabine (50 mg/kg) or control was given intraperitoneally twice a week for 30 days. Tumor growth was monitored by vernier caliper measurement every 3 days and the tumor volume was calculated according to the following formula: volume = (longest diameter × shortest diameter^2^)/2. Mice were sacrificed on day 30 after treatment. Tumors were excised and paraffin-embedded. The tissue sections were used in immunohistochemistry to detect GATA1 and Bcl-XL expression. TUNEL assay was performed using one-step TRITC-labeled TUNEL assay kit (KeyGEN BioTECH). Paraffin-embedded tissue sections were deparaffinized, rehydrated, incubated with Proteinase K, reacted with TdT enzyme, and labeled with TRITC and DAPI according to the manufacture's protocol. Fluorescence images of stained tissue sections were collected under a laser confocal microscope. TUNEL-positive cells were detected and quantified to determine the apoptotic index.

### 2.13. Immunohistochemistry

Immunohistochemistry (IHC) of formalin-fixed paraffin-embedded samples was performed as described previously [27]. Rabbit anti-GATA1 and rabbit anti-Bcl-XL were used at dilutions of 1:1000 as primary antibodies for IHC. The expression of GATA1 and Bcl-XL was determined by H score method. H score was generated by multiplying the percentage of stained cells (0-100%) by the intensity of the staining (low, 1+; medium, 2+; strong, 3+). Thus, the score is between 0 and 3. The median score was used to categorize low and high expression groups. We defined ≤1.215 as low GATA1 and ≤1.815 as low Bcl-XL.

### 2.14. Statistical Analysis

Differences between groups were compared using student's* t* test if a normal distribution is satisfied; otherwise, the nonparametric Mann-Whitney* U* test was applied. For multiple comparisons, one-way ANOVA was adopted. Repeated measures ANOVA were used to compare cell proliferation and viability curves and tumor growth curves. For survival and recurrence analysis, Kaplan-Meier method was conducted with the log-rank test. The Cox regression model was used to perform univariate and multivariate analyses. The correlation between GATA1 and Bcl-XL scores was verified by Spearman rank correlation analysis. By the correlation between clinical characteristics and GATA1, Bcl-XL expression was evaluated by Fisher exact test. All statistical analyses were performed using SPSS 23.0. All statistical tests were two-sided and* P *value <0.05 was considered statistically significant.

## 3. Results

### 3.1. GATA1 Is Highly Expressed in PDAC

**Figure 1:**
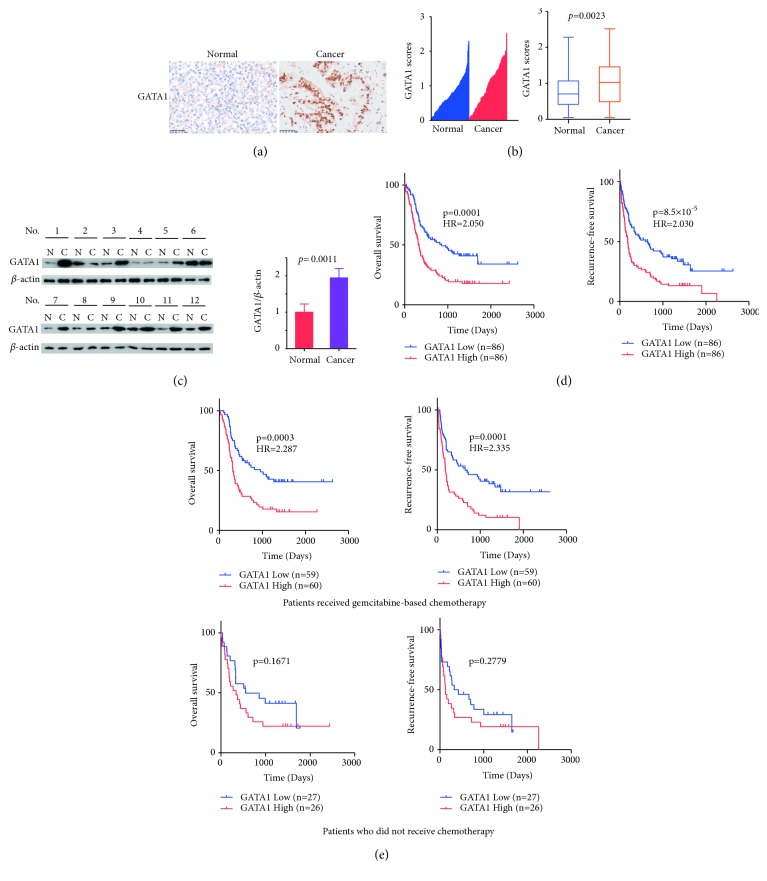
*GATA1 is overexpressed in PDAC and confers resistance to gemcitabine in PDAC patients*. (a) Representative immunohistochemical staining of GATA1 in 86 pairs of PDAC tissues and adjacent normal pancreas tissues. Scale bar: 50 *μ*m. (b) H scores of GATA1 expression between normal and cancer tissues were compared by Mann-Whitney* U* test. (c) Representative Western blot assay of tumor tissues and paired adjacent normal tissues. Paired* t* test was used to compare GATA1 expression between normal tissues and cancer tissues (n = 30). (d) Kaplan-Meier survival curves for overall survival and recurrence-free survival of 172 PDAC patients according to the relative expression of GATA1. (e) Kaplan-Meier survival curves of patients with (n = 119) or without gemcitabine treatment (n = 53).

To explore the clinical significance of GATA1 in PDAC, we performed immunohistochemistry (IHC) to determine GATA1 protein expression in 86 pairs of PDAC and matched paracancerous tissues. GATA1 antibody specificity was confirmed with antigen competition and Western blot of lysates from CFPAC-1 and PANC-1 cells stably expressed GATA1 shRNA (Figures S1a and S1b). Compared with paracancerous tissue, GATA1 expression was upregulated in PDAC tissues (P =0.0023) (Figures 1(a) and 1(b)). We further confirmed the upregulation of GATA1 in additional 30 pairs of fresh frozen PDAC tissues and matched paracancerous tissues with Western blot analysis (P =0.0011) (Figure 1(c)). These findings suggest that GATA1 expression is highly elevated in PDAC.

### 3.2. GATA1 Is an Independent Prognostic Factor in PDAC and Predicts Clinical Outcomes of Gemcitabine Therapy

We detected the correlation of GATA1 expression with clinical characteristics in a total of 172 PDAC patients. We observed that patients with highly expressed GATA1 had shorter OS (P=0.0001) and RFS (P=8.5×10^−5^) (Figure 1(d)). Since gemcitabine is the first-line drug for PDAC, we determined the correlation of GATA1 status with gemcitabine resistance in PDAC. In 172 PDAC patients, 119 of them received gemcitabine treatment. For these patients, those with highly expressed GATA1 showed poorer prognosis for OS (*P* = 0.0003) and RFS (*P *=0.0001) than those with low expressed GATA1. In contrast, the remaining 53 patients who did not receive any chemotherapy demonstrated no significant difference in their RFS and OS regardless of the GATA1 status (Figure 1(e)). Moreover, the univariate and multivariate analyses revealed that grade, node metastasis, vessels invasion, and GATA1 status were independent prognostic factors of OS and RFS (Tables S1 and S2). GATA1 expression positively associated with grade and vessels invasion, and but it did not associate with sex, age, and other clinical factors (Table S3). Taken together, these results indicated the significance of GATA1 in prognosis and response to gemcitabine treatment in PDAC.

### 3.3. GATA1 Promotes Cell Proliferation and Confers Gemcitabine Resistance in Pancreatic Cancer Cells

**Figure 2:**
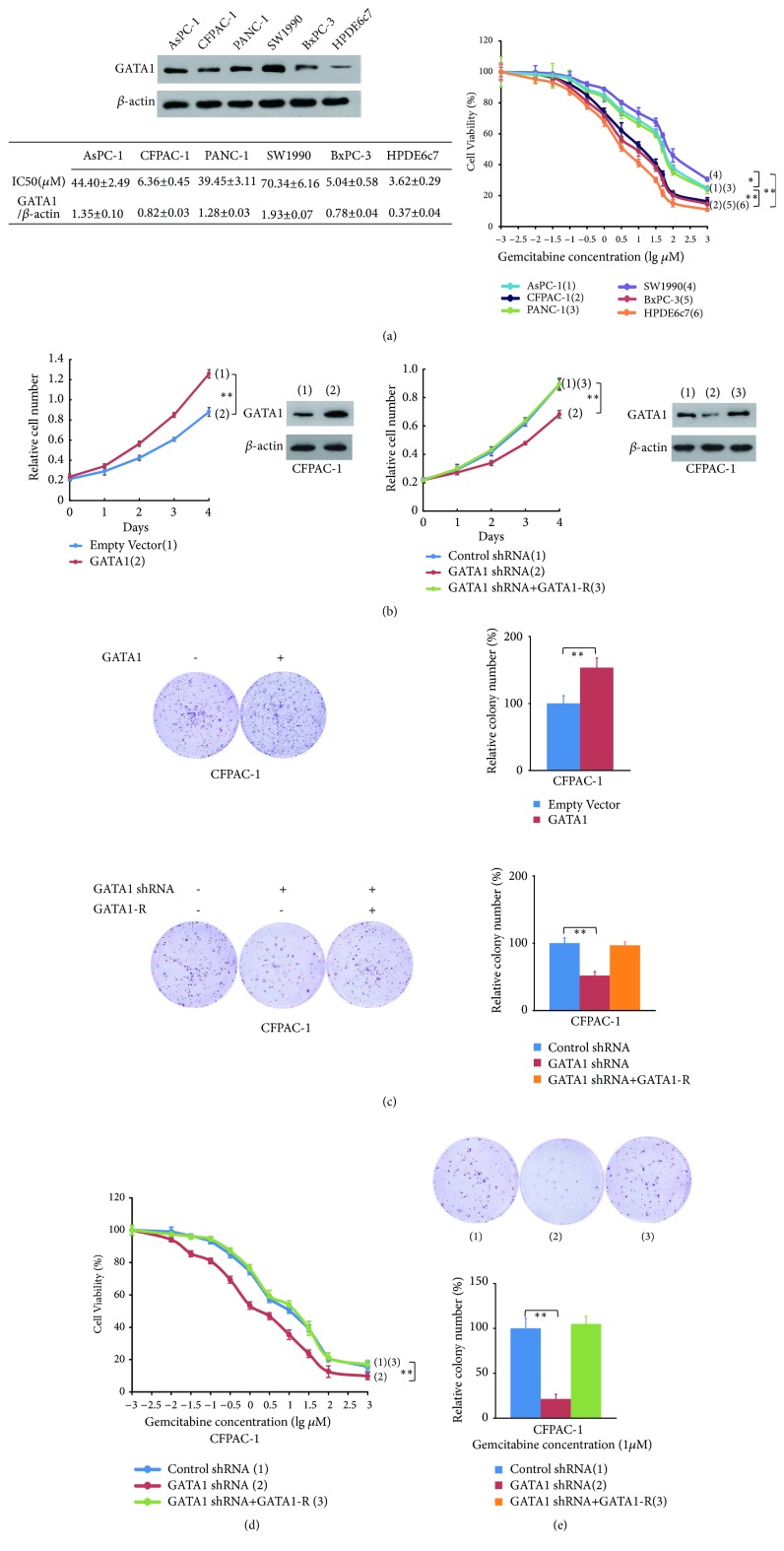
*GATA1 promotes cell proliferation and gemcitabine resistance in vitro.* (a) Western blot assay of GATA1 in PDAC and normal pancreatic cell lines. Relative expression of GATA1 in PDAC cell lines was quantified and listed in the table below along with the IC50 value. The cell viability curves of different PDAC cell lines were shown in the right panel. (b) Cell proliferation curves of CFPAC-1 cells stably infected with lentivirus carrying GATA1 (left panel), GATA1 shRNA, or GATA1 shRNA plus GATA1-R (right panel). The rescued cell line was established by reexpression of shRNA-resistant GATA1 (GATA1-R) in the GATA1 knockdown cells. GATA1 overexpression and knockdown effects in CFPAC-1 cells were validated by Western blot assay with *β*-actin as a loading control. (c) Representative images of colony formation assay in stable CFPAC-1 cells as established in (b). Relative colony numbers were quantified and compared by* t* test. (d) Cell viability assay of stable CFPAC-1 cells from (b). Cells were treated with a range of concentration of gemcitabine for 48 h before CCK8 test. (e) Representative images of colony formation assay in stable GATA1 knockdown CFPAC-1 cells. Cells were treated with gemcitabine (1 *μ*M) for 48 h. Relative colony numbers were quantified and compared by* t* test.

Based on previous observation, we further determined the effect of the GATA1 on proliferation and gemcitabine resistance of PDAC cells. First of all, we explored the GATA1 status and gemcitabine sensitivity in PDAC cell lines. Compared to GATA1 high expression cell lines (SW1990, AsPC-1, and PANC-1), the GATA1 low expression ones (CFPAC-1, BxPC-3, and HPDE6c7) are more sensitive to gemcitabine (Figure 2(a)). Subsequently, we established stable GATA1 overexpression and knockdown cell lines. GATA1 overexpression promoted cell proliferation in CFPAC-1 and PANC-1. On the contrary, GATA1 knockdown reduced cell proliferation, and GATA1 reexpression in the knockdown cells rescued this effect (Figures 2(b) and S2a). Consistent with the cell proliferation results, GATA1 overexpression in CFPAC-1 and PANC-1 cells increased cell colony formation. GATA1 knockdown decreased cell colony formation, and reexpression of GATA1 abolished this effect (Figures 2(c) and S2b). Afterwards, we investigated the effect of GATA1 on gemcitabine resistance in pancreatic cancer cells. Compared to control cells (IC50 value, CFPAC-1: 6.33 *μ*M; PANC-1: 37.88 *μ*M), GATA1 knockdown decreased the IC50 value of CFPAC-1 (IC50 value: 1.61 *μ*M) and PANC-1 cells (IC50 value: 9.72 *μ*M), and the effect was abolished by GATA1 reexpression (IC50 value, CFPAC-1: 6.25 *μ*M, PANC-1: 36.77 *μ*M), suggesting GATA1 knockdown increased sensitivity to gemcitabine, and this phenotype was reversed by GATA1 reexpression (Figures 2(d) and S2c).

Colony formation assay confirmed these results (Figures 2(e) and S2d). To sum up, GATA1 is critical for proliferation and intrinsic gemcitabine resistance in pancreatic cancer cells.

### 3.4. Screening for Target Genes Contributing to GATA1-Mediated Gemcitabine Resistance

**Figure 3:**
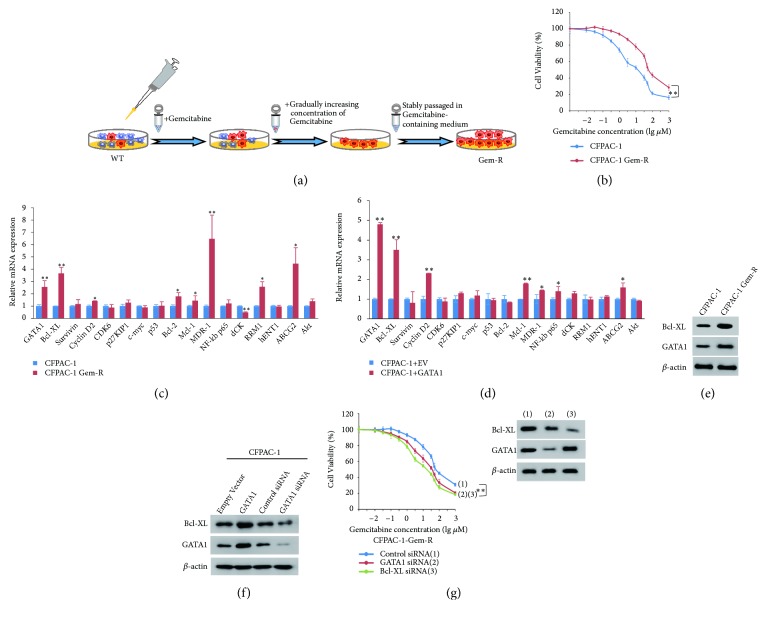
*Screening for target genes in GATA1 mediated gemcitabine resistance*. (a) Schematic representation of the protocol to obtain gemcitabine-resistant PDAC cell line (Gem-R). Red and blue colors of cells represent gemcitabine resistance and sensitivity, respectively. Gem-R cells were stably passaged for more than 10 times after exposed to an increasing concentration of gemcitabine for over 3 months. (b) Cell viability assays of CFPAC-1 and CFPAC-1 Gem-R cells treated with a range of concentration of gemcitabine for 48 h. (c) qRT-PCR analysis of relative mRNA expression in CFPAC-1 versus CFPAC-1 Gem-R cells of genes related to proliferation and drug resistance. (d) qRT-PCR analysis of relative mRNA expression in CFPAC-1 cells transiently transfected with empty vector or GATA1. The genes from (c) were used for qRT-PCR. (e) Elevated expression of GATA1 and Bcl-XL was detected in CFPAC-1 Gem-R by Western blot analysis. (f) Western blot analysis of Bcl-XL and GATA1 in CFPAC-1 cells transiently transfected with empty vector or GATA1, control siRNA, or GATA1 siRNA. *β*-actin was used as a loading control. (g) Cell viability curves of CFPAC-1-Gem-R cells transfected with GATA1 siRNA or Bcl-XL siRNA. The cells were exposed to a range of concentration of gemcitabine for 48 h before CCK8 assay. The knockdown effects of siRNAs were confirmed by Western blot analysis, with *β*-actin as a loading control. All data shown are means ± SD of three independent experiments with triplicate each, ^*∗*^*P* < 0.05; ^*∗∗*^*P* < 0.01.

To determine the implication of GATA1 in gemcitabine resistance, we established gemcitabine-resistant (Gem-R) CFPAC-1 and PANC-1 cell lines as acquired chemoresistant model through chronic gemcitabine exposure (Figure 3(a)). We confirmed that Gem-R cells (IC50 value, CFPAC-1: 33.86 *μ*M, PANC-1: 146.59 *μ*M) were more resistant to gemcitabine than the parental cells (IC50 value, CFPAC-1: 6.27 *μ*M; PANC-1: 38.01 *μ*M) with cell viability assay (Figures 3(b) and S3a). Next, we analyzed the mRNA expression of chemoresistance-related genes with Gem-R cells. Consistent with previous reports, Bcl-XL, cyclin D2, Bcl-2, Mcl-1, MDR-1, RRM1, and ABCG2 were upregulated, and dCK was downregulated. Intriguingly, GATA1 expression level was also elevated in Gem-R cells (Figures 3(c) and S3b). Next, we investigated the genes related to GATA1-mediated gemcitabine resistance. In CFPAC-1 and PANC-1 cells, GATA1 overexpression greatly improved Bcl-XL level and modestly enhanced cyclin D2, Mcl-1, MDR-1, NF-*κ*b p65, and ABCG2 expression (Figures 3(d) and S3c). Consistent with mRNA expression level, GATA1 and Bcl-XL protein level were also enhanced in Gem-R cells (Figures 3(e) and S3d). Further Western blot assay showed that GATA1 overexpression enhanced Bcl-XL level, and GATA1 knockdown decreased Bcl-XL expression (Figures 3(f) and S3e). Given that GATA1 and Bcl-XL were both enhanced in Gem-R cells, we performed cell viability assay to investigate the effect of GATA1 or Bcl-XL knockdown on gemcitabine resistance in Gem-R cells. The IC50 values of GATA1 knockdown cells (CFPAC-1: 17.55 *μ*M; PANC-1: 64.12 *μ*M) and Bcl-XL knockdown cells (CFPAC-1: 7.72 *μ*M; PANC-1: 49.75 *μ*M) were greatly downregulated compared to control cells (CFPAC-1: 30.88 *μ*M; PANC-1: 153.67 *μ*M), suggesting GATA1 and Bcl-XL knockdown both sensitized Gem-R cells to gemcitabine treatment (Figures 3(g) and S3f). These results suggest that GATA1 was involved in gemcitabine acquired resistance through Bcl-XL in PDAC cells.

### 3.5. GATA1 Regulates Bcl-XL Transcription through Binding to Its Promoter in Pancreatic Cancer Cells

**Figure 4:**
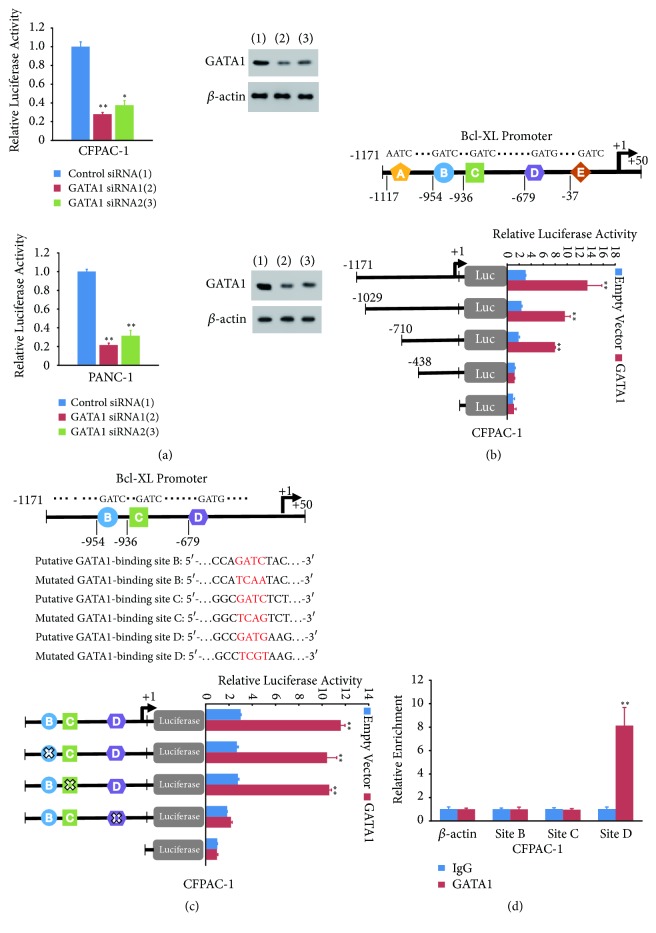
*GATA1 regulates Bcl-XL transcription through binding to its promoter*. (a) Luciferase reporter assay of CFPAC-1 and PANC-1 cells cotransfected with the Bcl-XL promoter-Luc reporter and control siRNA or GATA1 siRNAs. Representative Western blot analysis indicates the expression of GATA1. (b) Relative luciferase activity of different truncated Bcl-XL promoter reporter constructs in CFPAC-1 cells transfected with empty vector or GATA1. A, B, C, D, and E indicate putative binding sites of GATA1. (c) Relative luciferase activity of wild-type and mutated Bcl-XL promoter reporter constructs in CFPAC-1 cells transfected with empty vector or GATA1. B, C, and D indicate putative binding sites of GATA1. The “X” symbol denotes mutated GATA1-binding sites. (d) ChIP analysis of the occupancy of GATA1 protein on putative GATA1-binding sites. All values shown are means ± SD of three independent experiments with triplicate each, ^*∗*^*P* < 0.05; ^*∗∗*^*P* < 0.01 versus corresponding control.

Since GATA1 improved Bcl-XL mRNA and protein level, we investigated whether GATA1 regulates Bcl-XL expression through binding to its promoter. First of all, we detected the effect of GATA1 on the transcriptional activity of Bcl-XL promoter (from –1171 to +50 bp)-luciferase. Knockdown of GATA1 greatly inhibited the transcriptional activity of Bcl-XL promoter, suggesting GATA1 bound to Bcl-XL promoter (Figure 4(a)). We then determined the binding site of GATA1 on Bcl-XL promoter. Actually, there are five putative binding sites of GATA1 on Bcl-XL promoter (from –1171 to +50 bp) (Figure 4(b)). Truncated promoter reporters were used to identify the binding site of GATA1 on Bcl-XL promoter. The deletion of site D containing GATG motif abolished GATA1-mediated enhancement of Bcl-XL promoter transcriptional activity, while deletion of the other three putative sites did not (Figures 4(b) and S4a). Mutated promoter reporter analysis confirmed that site D was responsible for GATA1 modulation of Bcl-XL promoter reporter activity, since GATA1 increased the activity of the reporter with the mutated site B or C, but not with the mutated site D in CFPAC-1 and PANC-1 cells (Figures 4(c) and S4b). Moreover, chromatin immunoprecipitation assay (ChIP) assay showed that GATA1 was recruited to the region containing site D, but not the region containing site B and C (Figure 4(d)). In conclusion, these results suggest that GATA1 binds to Bcl-XL promoter (-679/-676) to regulate its transcription.

### 3.6. GATA1 Inhibited Gemcitabine-Induced Apoptosis through Bcl-XL In Vitro

**Figure 5:**
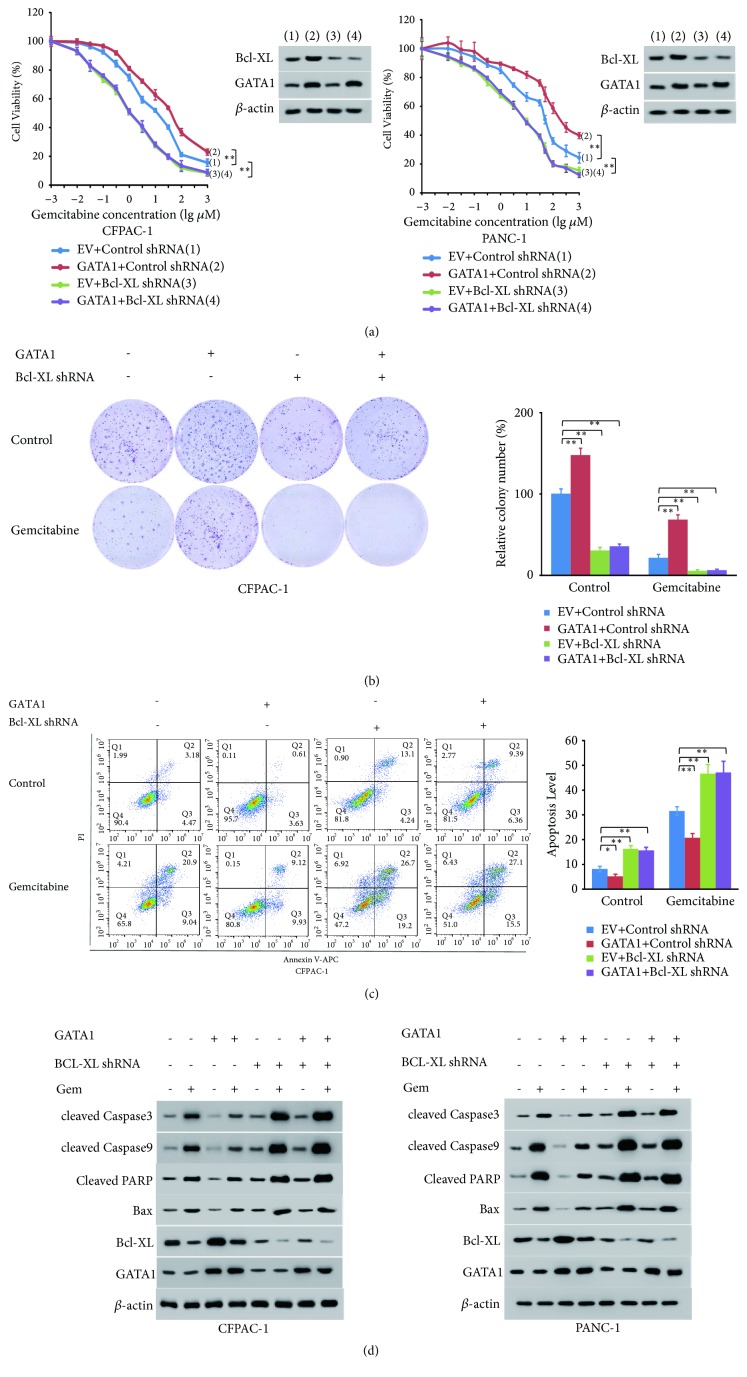
*GATA1 inhibits gemcitabine-induced apoptosis through Bcl-XL in vitro*. (a) Cell viability curves of CFPAC-1 and PANC-1 cells stably infected with the indicated lentiviruses. Western blot assay of GATA1 and Bcl-XL expression in indicated cells. *β*-actin was used as a loading control. Cells were treated with a range of concentration of gemcitabine for 48 h before CCK8 assay. (b) Representative images of colony formation assays in stable CFPAC-1 cells lines from (a). The cells were treated with control or gemcitabine (1 *μ*M) for 48 h before seeded into 6-well plates. Relative colony numbers were quantified and compared by* t* test. (c) Representative images of flow cytometry analysis of apoptosis in CFPAC-1 cells transfected with indicated lentivirus. The cells were treated with or without gemcitabine (5 *μ*m) for 48 h. Statistical analysis of apoptosis rates was shown in the right panel. (d) Western blot analysis of GATA1, Bcl-XL, Bax, cleaved PARP, cleaved caspase 9, and cleaved caspase 3 in CFPAC-1 and PANC-1 infected with the indicated lentivirus. The cells were treated with or without gemcitabine (10 *μ*M for CFPAC-1 and 50 *μ*M for PANC-1) for 48 h before analysis. All data shown are means ± SD of three independent experiments with triplicate each, ^*∗*^*P* < 0.05; ^*∗∗*^*P* < 0.01.

Since GATA1 and Bcl-XL are both involved in gemcitabine resistance of pancreatic cancer cells, and Bcl-XL is a target gene of GATA1, we tested whether GATA1 promotes gemcitabine resistance through regulating Bcl-XL level with cell viability assay and colony formation assay. GATA1 overexpression upregulated the IC50 value of CFPAC-1 (6.3 *μ*M to 17.55 *μ*M) and PANC-1 cells (42.431 *μ*M to 111.74 *μ*M), whereas Bcl-XL knockdown inhibited the IC50 value (CFPAC-1: 0.915 *μ*M and PANC-1: 6.858 *μ*M) of the cells, and Bcl-XL knockdown abolished the upregulation induced by GATA1 overexpression (CFPAC-1: 0.979 *μ*M and PANC-1: 8.408 *μ*M). The IC50 values indicated that GATA1 overexpressed cells displayed higher resistance to gemcitabine, and the knockdown of Bcl-XL reversed this effect (Figures 5(a), 5(b), and S5a). In the PDAC cells without gemcitabine treatment, GATA1 overexpression inhibited apoptosis, and knockdown of Bcl-XL effectively abrogated this effect, suggesting GATA1 inhibited endogenous apoptosis by Bcl-XL. Importantly, GATA1 greatly inhibited gemcitabine-induced apoptosis of PDAC cells, and Bcl-XL knockdown abolished this effect, suggesting GATA1 mediated gemcitabine resistance through Bcl-XL (Figures 5(c) and S5b). Moreover, we detected the expression of apoptotic proteins with Western blot analysis. Decreased expression of Bax, cleaved PARP, cleaved caspase-9, and cleaved caspase-3 was observed in GATA1 overexpressed cells, while elevated expression was observed in Bcl-XL knockdown cells (lanes 1, 3, and 5). Gemcitabine greatly enhanced the expression of these apoptotic proteins, and GATA1 remarkably offset this effect (lanes 1, 2, and 4). Downregulation of Bcl-XL abolished the effect of GATA1 on these apoptotic proteins no matter with or without gemcitabine treatment (lane 5-8) (Figures 5(d) and S5c). In conclusion, GATA1 decreased endogenous and gemcitabine induced apoptosis through Bcl-XL.

### 3.7. GATA1 Mediates Gemcitabine Resistance of PDAC through Bcl-XL In Vivo

**Figure 6:**
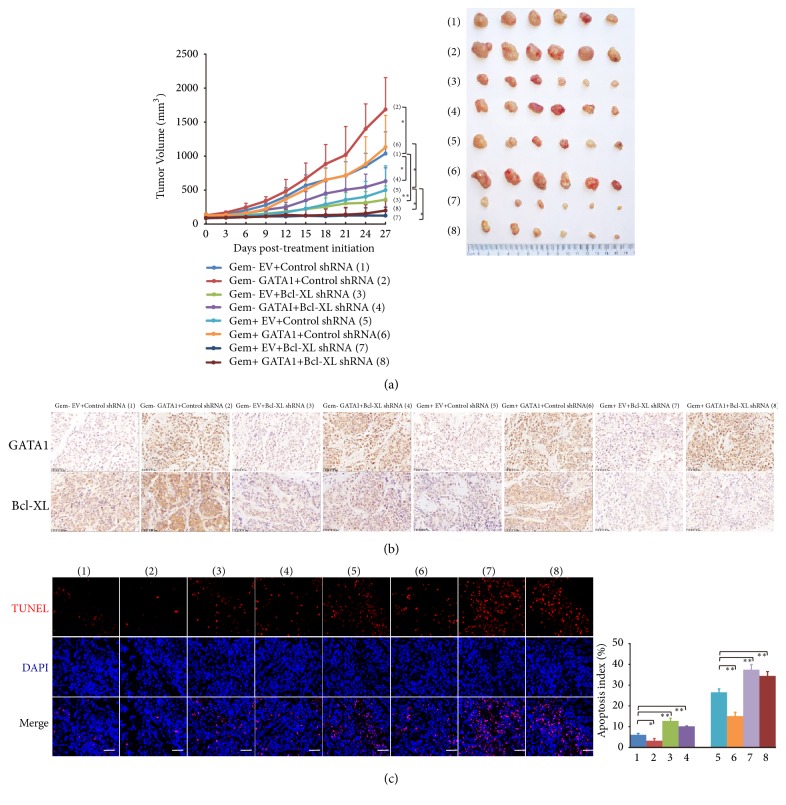
*GATA1 confers gemcitabine resistance of PDAC through Bcl-XL in vivo*. (a) Volume of xenografts tumors derived from PANC-1 cells infected with the indicated lentivirus and treated with gemcitabine or control. The tumors were measured by vernier caliper every 3 days and the tumor growth curves were plotted. Tumor volumes were presented as means ± SD (n = 6). Images of all xenografts tumors excised at day 27 after treatment were shown in the right. ^*∗*^*P* < 0.05; ^*∗∗*^*P* < 0.01. (b) Representative immunohistochemical staining of GATA1 and Bcl-XL in xenografts tumors of the indicated groups from (a). Scale bar: 50 *μ*m. (c) Representative images of apoptotic cells visualized by tunnel staining and counterstained by DAPI in tumor sections of the indicated groups from (a). Scale bar: 50 *μ*m. Apoptosis index was quantified and analyzed. Data are shown in means ± SD. ^*∗*^*P* < 0.05; ^*∗∗*^*P* < 0.01.

We further confirmed GATA1 mediated gemcitabine resistance with xenograft mouse model. GATA1 overexpression markedly promoted tumor growth, whereas knockdown of Bcl-XL dramatically inhibited pancreatic tumor growth, and Bcl-XL knockdown offset GATA1 mediated tumor growth. Upon the treatment of gemcitabine, we observed that GATA1 greatly counteracted gemcitabine-induced tumor shrink. However, Bcl-XL knockdown completely abolished GATA1 conferred gemcitabine resistance (Figure 6(a)). IHC assay verified the overexpression of GATA1 and knockdown of Bcl-XL in xenograft tumors (Figure 6(b)). TUNEL assay was used to determine the apoptosis index of xenograft tumors. GATA1 overexpression decreased the proportion of TUNEL-positive cells, and Bcl-XL knockdown increased the ratio of TUNEL-positive cells. Moreover, the effect of GATA1 on apoptotic index was abrogated by Bcl-XL knockdown. Gemcitabine greatly enhanced the proportion of TUNEL-positive cells, and GATA1 overexpression decreased gemcitabine-induced apoptosis significantly, whereas knockdown of Bcl-XL abrogated the antiapoptotic effect of GATA1 (Figure 6(c)). The results need to be confirmed with orthotopic nude mouse model. Taken together, GATA1 mediates gemcitabine resistance through Bcl-XL related antiapoptosis in* vivo*.

### 3.8. Bcl-XL Positively Correlates with GATA1 and Is a Prognostic Marker for Gemcitabine Treatment in PDAC

**Figure 7:**
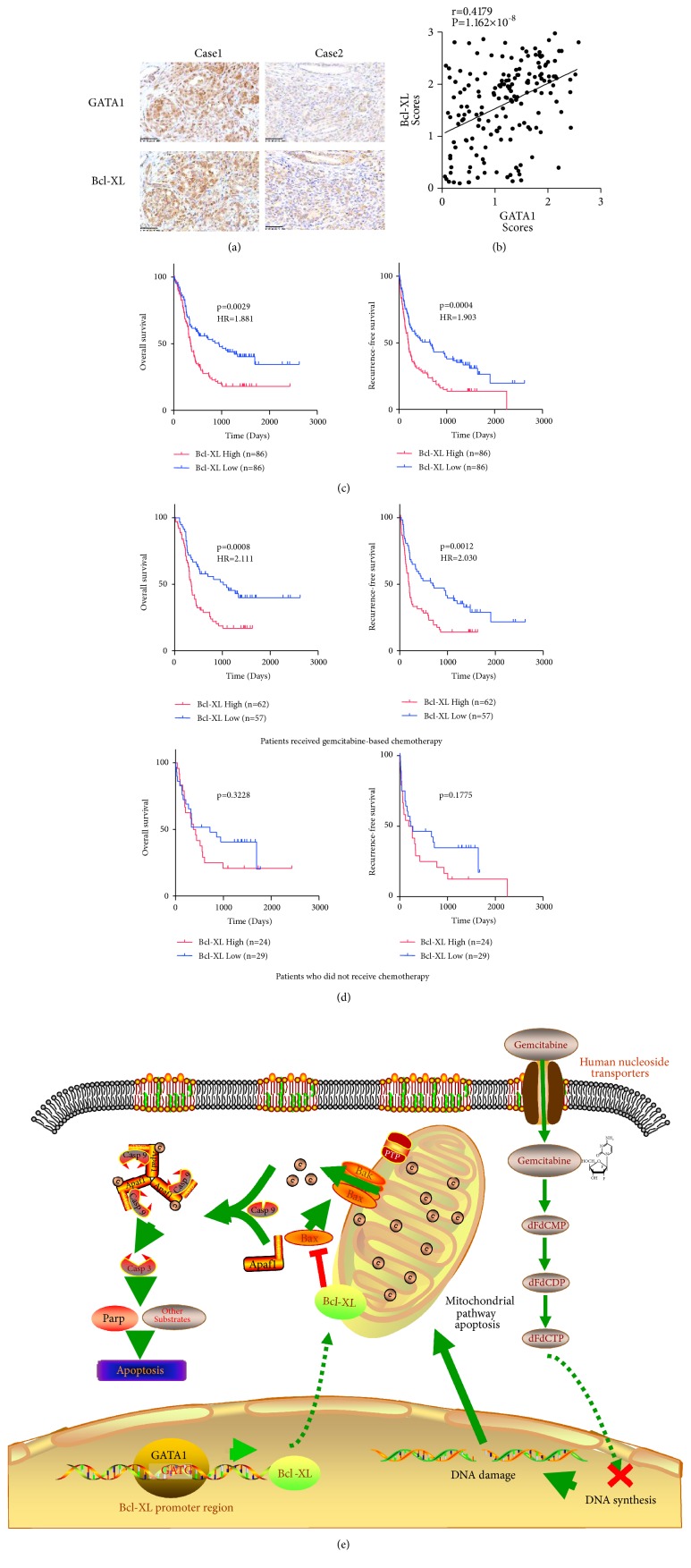
*Bcl-XL positively correlates with GATA1 and is a prognostic marker for PDAC*. (a) Representative immunohistochemical staining of GATA1 and Bcl-XL in human PDAC tissues. Scale bar: 50 *μ*m. (b) Correlation between GATA1 and Bcl-XL expression was analyzed in 172 PDAC samples (from Figure 1(d)) by Spearman rank correlation analysis. (c) Kaplan-Meier survival curves for overall survival and recurrence-free survival of 172 PDAC patients (from Figure 1(d)) according to the relative expression of Bcl-XL. (d) Kaplan-Meier survival curves of patients with (n=119) or without gemcitabine treatment (n = 53). (e) Graphic summary of GATA1 promoting gemcitabine resistance through antiapoptotic pathway. Gemcitabine causes apoptosis in mitochondrial pathway owing to DNA synthesis inhibition followed by DNA damage. GATA1 acts as a transcription activator by binding to the “GATG” site of Bcl-XL promoter region, leading to upregulation of Bcl-XL expression. Elevated Bcl-XL expression can counteract gemcitabine-induced apoptosis via binding to Bax, preventing the release of cytochrome c and thus preventing caspase activation.

As GATA1 binds to Bcl-XL promoter and regulates Bcl-XL expression, we subsequently determined the correlation of GATA1 and Bcl-XL in PDAC samples. We verified the specificity of the Bcl-XL antibody used in IHC by antigen competition and lysates of Bcl-XL knockdown cells (Figures S6a and S6b). The IHC assay indicated that expression of GATA1 positively correlated with Bcl-XL expression (Figures 7(a) and 7(b)). PDAC patients with high Bcl-XL expression had shorter OS (*P*=0.0029) and RFS (*P*=0.0004) (Figure 7(c)). Next, we detected the effect of Bcl-XL expression on gemcitabine response in PDAC. For patients who received gemcitabine-based chemotherapy, those with highly expressed Bcl-XL showed poorer prognosis for OS (*P* = 0.0008) and RFS (*P *=0.0012) than those with low Bcl-XL expression. In contrast, patients who did not receive any chemotherapy demonstrated no significant difference in their RFS and OS between high and low Bcl-XL expression (Figure 7(d)). Moreover, Bcl-XL status was proved to be an independent prognostic factor according to the univariate and multivariate analyses (Tables S1 and S2). In conclusion, these data indicated that Bcl-XL has a major role in the resistance of gemcitabine treatment in PDAC.

## 4. Discussion

Gemcitabine remains a cornerstone for PDAC treatment since 1997; however, the intrinsic and acquired resistance to this chemotherapy inhibits its function and leads to poor outcome of patients [28]. Although multiple mechanisms of gemcitabine resistance have been reported, no significant advances have been achieved to improve the prognosis of PDAC patients. Therefore, a novel target to enhance current chemotherapy is clearly needed to improve the outcomes of patients with pancreatic cancer.

In the current study, we identified a novel role of GATA1 in tumor proliferation and gemcitabine resistance in PDAC. Firstly, we found that, compared to normal pancreatic tissue, GATA1 was overexpressed in PDAC tissues, and the expression of GATA1 was correlated with overall survival, recurrence-free survival, and gemcitabine response. Secondly, GATA1 upregulated Bcl-XL transcription by binding to its promoter. Thirdly, GATA1 induced intrinsic and acquired resistance to gemcitabine in PDAC cells through enhancing Bcl-XL mediated antiapoptosis. Moreover, Bcl-XL expression was positively correlated with GATA1 and predicted clinical outcomes and gemcitabine response in PDAC patients. These findings indicated that GATA1 and Bcl-XL may be potential therapeutic targets to improve prognosis of PDAC patients.

Previous reports about GATA1 mainly focused on its function in erythroid and megakaryocytic cells. GATA1 regulates the differentiation, proliferation, and apoptosis of erythroid and megakaryocytic cells [23]. Mutation of GATA1 leads to acute megakaryoblastic leukemia (AMKL) in infants with Down syndrome and transient myeloproliferative disorder (TMD). Knockdown of GATA1 to 5% of its wild-type expression level causes high incidence of erythroid leukemia in mice [29]. Recently, more and more researches indicated that GATA1 played an important role in solid cancer. In breast cancer and colorectal cancer, GATA1 was reported to be overexpressed compared with matched adjacent normal tissues [24, 30]. Furthermore, GATA1 enhanced breast cancer cells invasion and angiogenesis through promoting epithelial-mesenchymal transition (EMT) [31] and VEGF expression [26]. Here, we found a novel function of GATA1 in gemcitabine resistance in pancreatic cancer. High expression of GATA1 is associated with poor prognosis of PDAC patients treated with gemcitabine, indicating GATA1 was correlated with gemcitabine resistance in PDAC patients. The PDAC cell lines (SW1990, AsPC-1, and PANC-1) that displayed relatively higher basal levels of GATA1 are also more resistant to gemcitabine. Based on these data, we may assume that GATA1 is correlated with intrinsic gemcitabine resistance. On the other hand, GATA1 expression was upregulated in established gemcitabine-resistant cell lines, suggesting GATA1 was also associated with acquired chemoresistance.

The activation of antiapoptotic genes might be responsible for acquired resistance of pancreatic cancer cells to gemcitabine. Mutated p53 confers resistance to gemcitabine as the mutation abolished its function in inhibiting Bcl-XL expression [32]. Downregulation of BNIP3, which could antagonize the activity of antiapoptotic proteins such as Bcl-2 and Bcl-XL and promote apoptosis, associated with intrinsic chemoresistance to gemcitabine in PDAC cells [33]. In this research, we elucidated a novel pathway of gemcitabine resistance through activating Bcl-XL. GATA1 enhanced Bcl-XL expression through binding to its promoter and then promoted Bcl-XL mediated antiapoptosis, leading to gemcitabine resistance in PDAC cells (Figure 7(e)). The levels of GATA1 and Bcl-XL were both upregulated in established gemcitabine-resistant cells. Moreover, Bcl-XL knockdown sensitized the Gem-R cells to gemcitabine treatment, suggesting Bcl-XL was involved in GATA1-induced acquired gemcitabine resistance. Also, Bcl-XL knockdown greatly sensitized the tumors to gemcitabine in* vivo*. However, the subcutaneous xenograft models could not fully mimic the physiological tumor development. The effect of Bcl-XL on gemcitabine resistance needs further confirmation with the orthotopic models. The high expression of both GATA1 and Bcl-XL correlates to poor clinical outcomes in patients treated with gemcitabine, suggesting GATA1 and Bcl-XL could be used as prognostic markers for gemcitabine treatment. Furthermore, GATA1 inhibition alone or in combination with Bcl-XL inhibition of may be a useful strategy for treating gemcitabine-resistant PDAC patients with high GATA1 level.

In addition to conferring gemcitabine resistance, GATA1 was also showed to promote cancer cell proliferation in* vitro* and in* vivo*. Mechanistically, cyclin D2, frequently thought to play a critical role in promoting tumor cell proliferation [34], was upregulated in GATA1 overexpressed cells. We speculate that GATA1 confers gemcitabine resistance in pancreatic cancer not only by activating Bcl-XL, but also by retaining proliferative potential of PDAC cancer following treatment with gemcitabine. In conclusion, cyclin D2 may play a substantial role in GATA1 mediated gemcitabine resistance.

In summary, we characterized the significance of GATA1/Bcl-XL axis in gemcitabine resistance in PDAC cells, and inhibition of this axis in PDAC cells enhanced the sensitivity of gemcitabine. Importantly, GATA1 and Bcl-XL both predict clinical outcomes of gemcitabine therapy. These findings might provide a promising strategy and target to develop new therapy against pancreatic cancer.

## 5. Conclusions

Taken together, we demonstrated that GATA1 is a new predictive marker for prognosis and gemcitabine resistance in pancreatic cancer patients. We found that GATA1 is involved in both intrinsic and acquired gemcitabine resistance in PDAC cells through enhancing Bcl-XL mediated antiapoptosis in vitro and in vivo. Furthermore, Bcl-XL expression is positively correlated with GATA1 and predicts clinical outcomes and gemcitabine response in PDAC patients.

## Data Availability

The data used to support the findings of this study are available from the corresponding author upon request.

## Supplementary Materials

Supplementary Materials Figure S1: validation of GATA1 antibody specificity; Figure S2: GATA1 overexpression promotes cell proliferation and confers gemcitabine resistance in vitro; Figure S3: screening of target genes responsible for GATA1-mediated gemcitabine resistance; Figure S4: GATA1 regulates Bcl-XL through binding to its promoter; Figure S5: GATA1 mediates gemcitabine resistance of PDAC through Bcl-XL; Figure S6: validation of Bcl-XL antibody specificity; Table S1: Cox univariate and multivariate analysis of overall survival in PDAC patients; Table S2: Cox univariate and multivariate analysis of recurrence-free survival in PDAC patients; Table S3: the relationship of GATA1 and Bcl-XL with clinical characteristics in PDAC patients; Table S4: sequences for shRNA, qRT-PCR, and ChIP primers.
