# Supplementary material for: GATA1 Promotes Gemcitabine Resistance in Pancreatic Cancer through Antiapoptotic Pathway
Source: J Oncol. 2019 Apr 10;2019:9474273. doi: 10.1155/2019/9474273 (PMC6481023; doi:10.1155/2019/9474273)
Supplement: Supplementary Materials — Figure S1: validation of GATA1 antibody specificity; Figure S2: GATA1 overexpression promotes cell proliferation and confers gemcitabine resistance in vitro; Figure S3: screening of target genes responsible for GATA1-mediated gemcitabine resistance; Figure S4: GATA1 regulates Bcl-XL through binding to its promoter; Figure S5: GATA1 mediates gemcitabine resistance of PDAC through Bcl-XL; Figure S6: validation of Bcl-XL antibody specificity; Table S1: Cox univariate and multivariate analysis of overall survival in PDAC patients; Table S2: Cox univariate and multivariate analysis of recurrence-free survival in PDAC patients; Table S3: the relationship of GATA1 and Bcl-XL with clinical characteristics in PDAC patients; Table S4: sequences for shRNA, qRT-PCR, and ChIP primers. [file 9474273.f1.zip › Supplementary Figures.pdf]

a

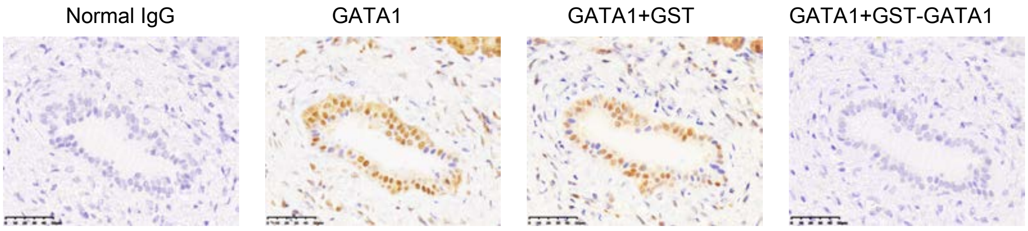

b

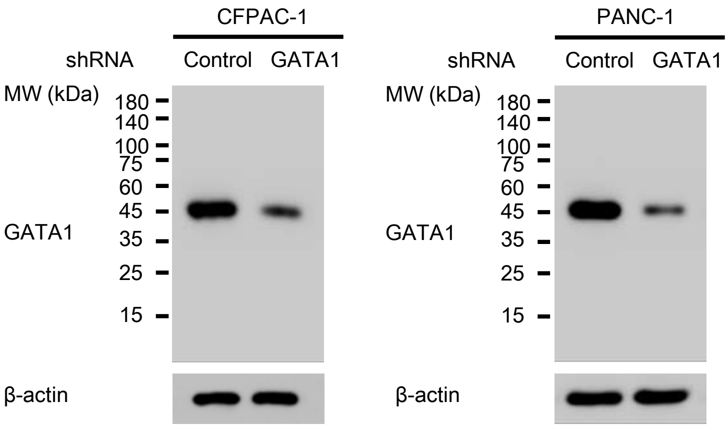

Figure S1 Validation of GATA1 antibody specificity.

a Immunohistochemical staining of human PDAC samples incubated with normal IgG or anti-GATA1. For validation of antibody specificity, The anti-GATA1 was incubated with recombinant GST-GATA1 or GST tag for 1 h before applying to tissue sections. Scale bar: 50 μm.

b Detection of GATA1 in CFPAC-1 and PANC-1 cell lysates stably infected with control shRNA or GATA1 shRNA by Western blot analysis with GATA1 antibody. MW: molecular weight.

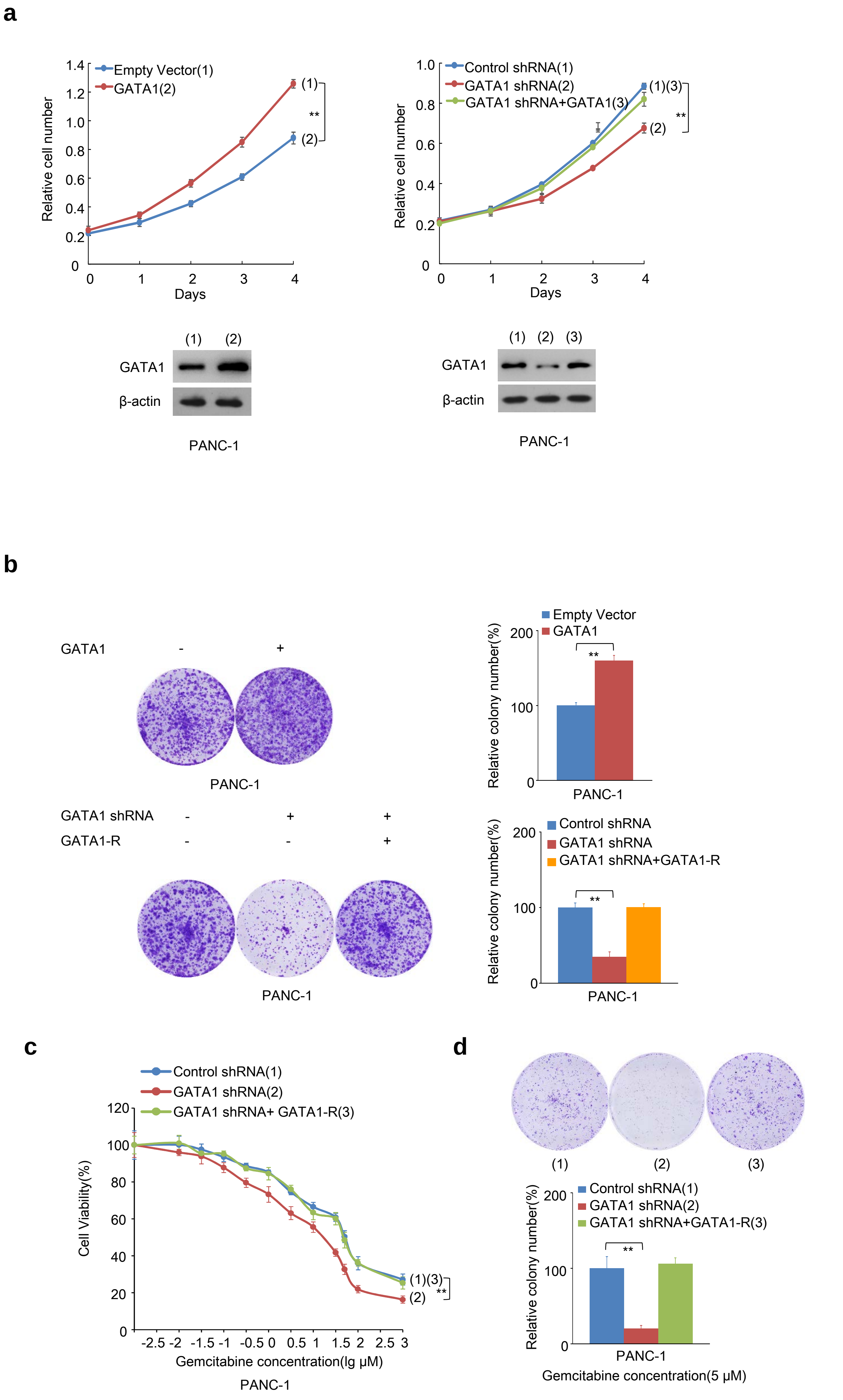

Figure S2 GATA1 overexpression promotes cell proliferation and confers gemcitabine resistance in *vitro*

a Cell proliferation curves of CFPAC-1 cells stably infected with lentivirus carrying GATA1 (Left Panel), GATA1 shRNA or GATA1 shRNA plus GATA1-R (Right Panel). The rescued cell line was established by reexpression of shRNA-resistant GATA1 (GATA1-R) in the GATA1 knockdown cells. GATA1 overexpression and knockdown effects in CFPAC-1 cells were validated by western blot assay with β-actin as a loading control. **b** Representative images of colony formation assays in PANC-1 cells stably infected as in A. Relative colony numbers were quantified and compared by *t* test. **c** Cell viability curves of PANC-1 cells stably infected with GATA1 shRNA or GATA1 shRNA plus GATA1-R. The cells were treated with a range of concentration of gemcitabine for 48 h before CCK8 test. **d** Representative images of colony formation assays in PANC-1 cells stably infected with the indicated lentivirus. Cells were treated with gemcitabine (5 μm) for 48 h before seeded into 6-well plates. Relative colony numbers were quantified and compared by *t* test. All data presented are means ± SD of three independent experiments with triplicate each, * $p < 0.05$, ** $p < 0.01$.

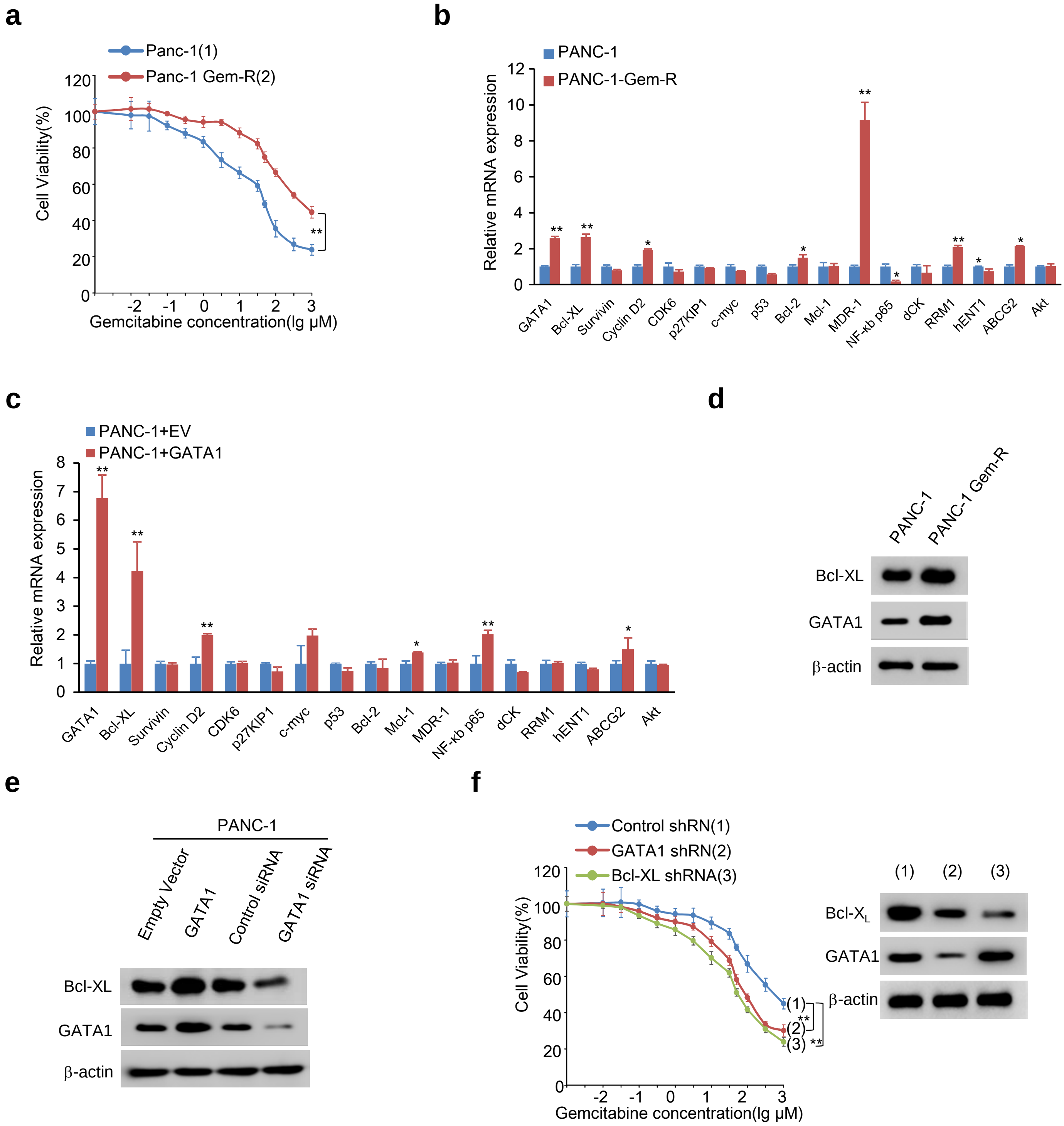

Figure S3 Screening of target genes responsible for GATA1-mediated gemcitabine resistance

a Cell viability assays of PANC-1 and PANC-1 Gem-R cells treated with a range of concentration of gemcitabine for 48 h before CCK8 test. **b** qRT-PCR analysis of relative mRNA expression in PANC-1 versus PANC-1 Gem-R cells of genes related to proliferation and drug resistance. **c** qRT-PCR analysis of relative mRNA expression in PANC-1 cells transiently transfected with empty vector or GATA1. The genes from B were used for qRT-PCR. **d** Elevated expression of GATA1 and Bcl-XL were detected in PANC-1 Gem-R by Western blot analysis**. e** Western blot analysis of Bcl-XL and GATA1 in PANC-1 cells transiently transfected with empty vector or GATA1, control siRNA or GATA1 siRNA. β-actin was used as a loading control. **f** Cell viability curves of PANC-1-Gem-R cells transfected with GATA1 siRNA or Bcl-XL siRNA. The cells were exposed to a range of concentration of gemcitabine for 48 h before CCK8 assay. The knockdown effects of siRNAs were confirmed by Western blot analysis, with β-actin as a loading control.

All data shown are means ± SD of three independent experiments with triplicate each, * $p < 0.05$, ** $p < 0.01$.

a

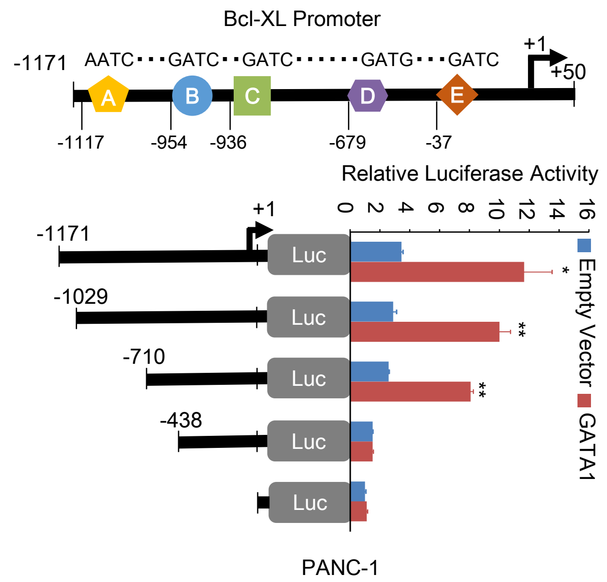

b

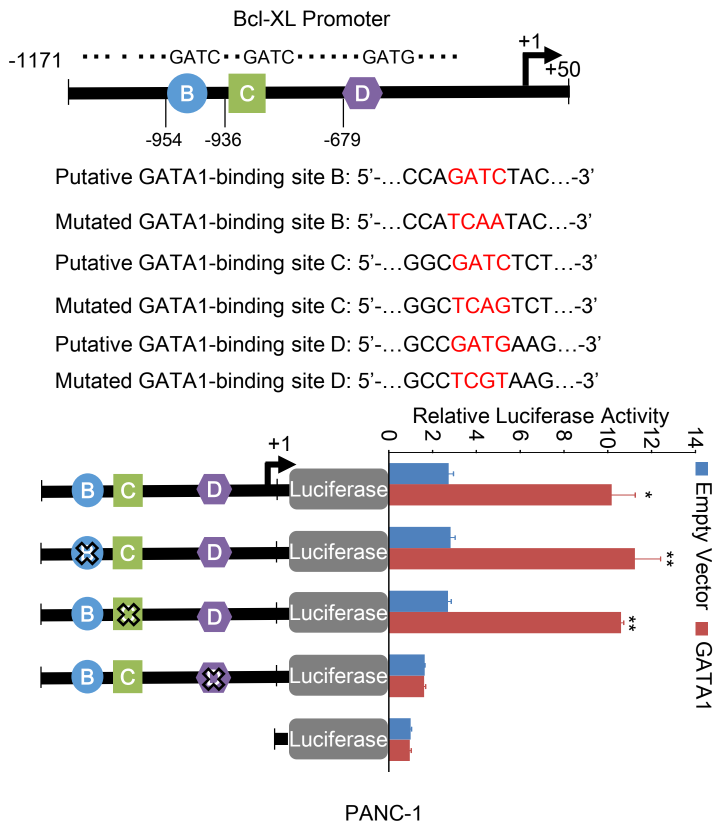

Figure S4 GATA1 regulates Bcl-XL through binding to its promoter
a Relative luciferase activity of different truncated Bcl-XL promoter reporters in PANC-1 cells transfected with empty vector or GATA1. A, B, C, D and E indicate putative binding sites of GATA1. **b** Relative luciferase activity of wild-type and mutated Bcl-XL promoter reporter constructs in PANC-1 cells transfected with empty vector or GATA1. B , C and D indicate putative binding sites of GATA1. The "X" symbol denotes mutated GATA1-binding sites. All values shown are means ± SD of three independent experiments with triplicate each, * $p < 0.05$, ** $p < 0.01$.

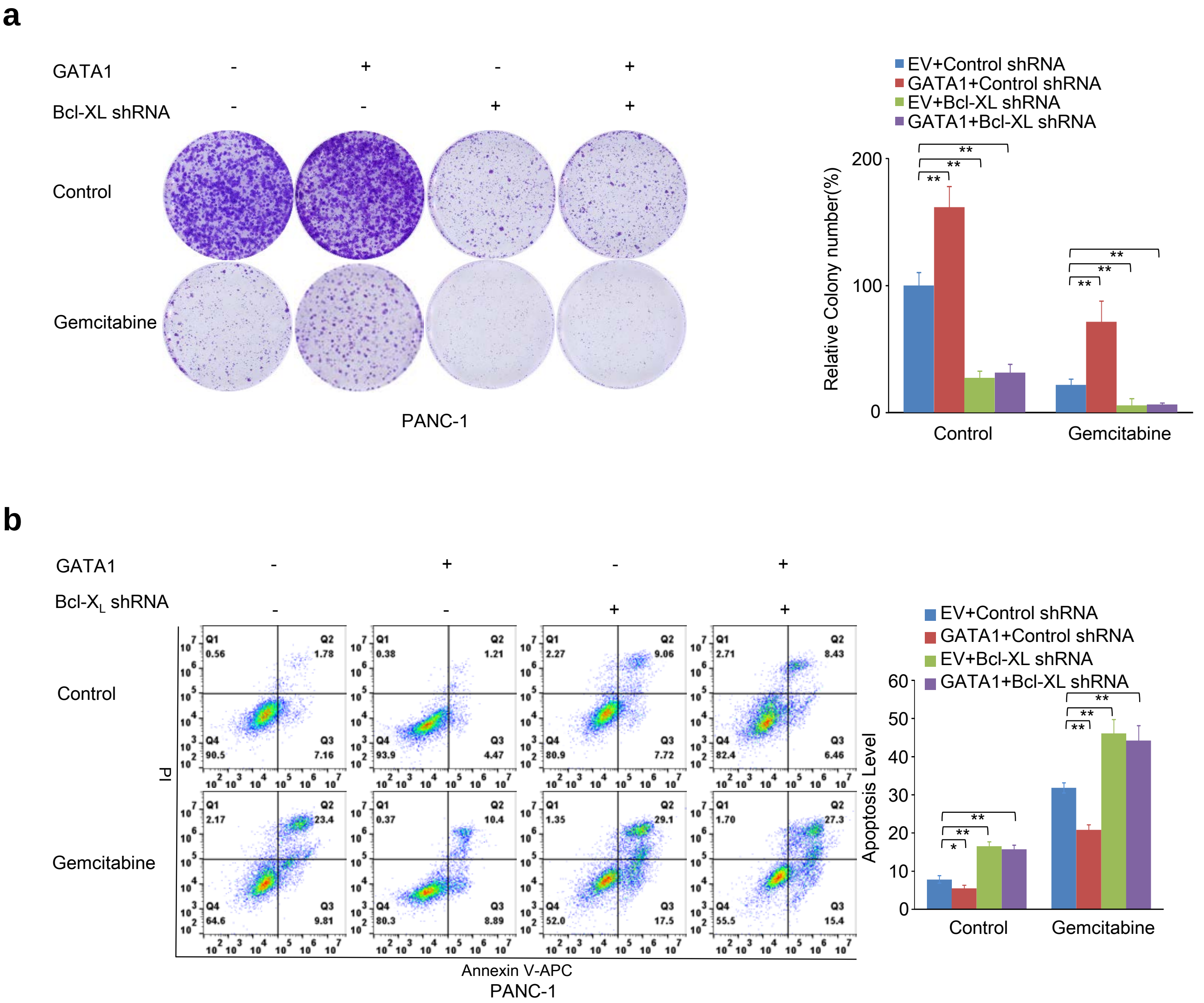

Figure S5 GATA1 mediates gemcitabine resistance of PDAC through Bcl-XL

a Representative images of colony formation assays in PANC-1 cells stably infected with the indicated lentivirus. Cells were treated with or without gemcitabine (5 μM) for 48 h before seeded into 6-well plates. Relative colony numbers were quantified and compared by *t* test. **b** Representative images of flow cytometry analysis of apoptosis in PANC-1 cells infected with the indicated lentivirus. Cells were treated with DMSO or gemcitabine (30 μM) for 48 h. Statistical analysis of apoptosis rates was shown in the right panel. All data shown are means ± SD of three independent experiments with triplicate each, * $p < 0.05$, ** $p < 0.01$.

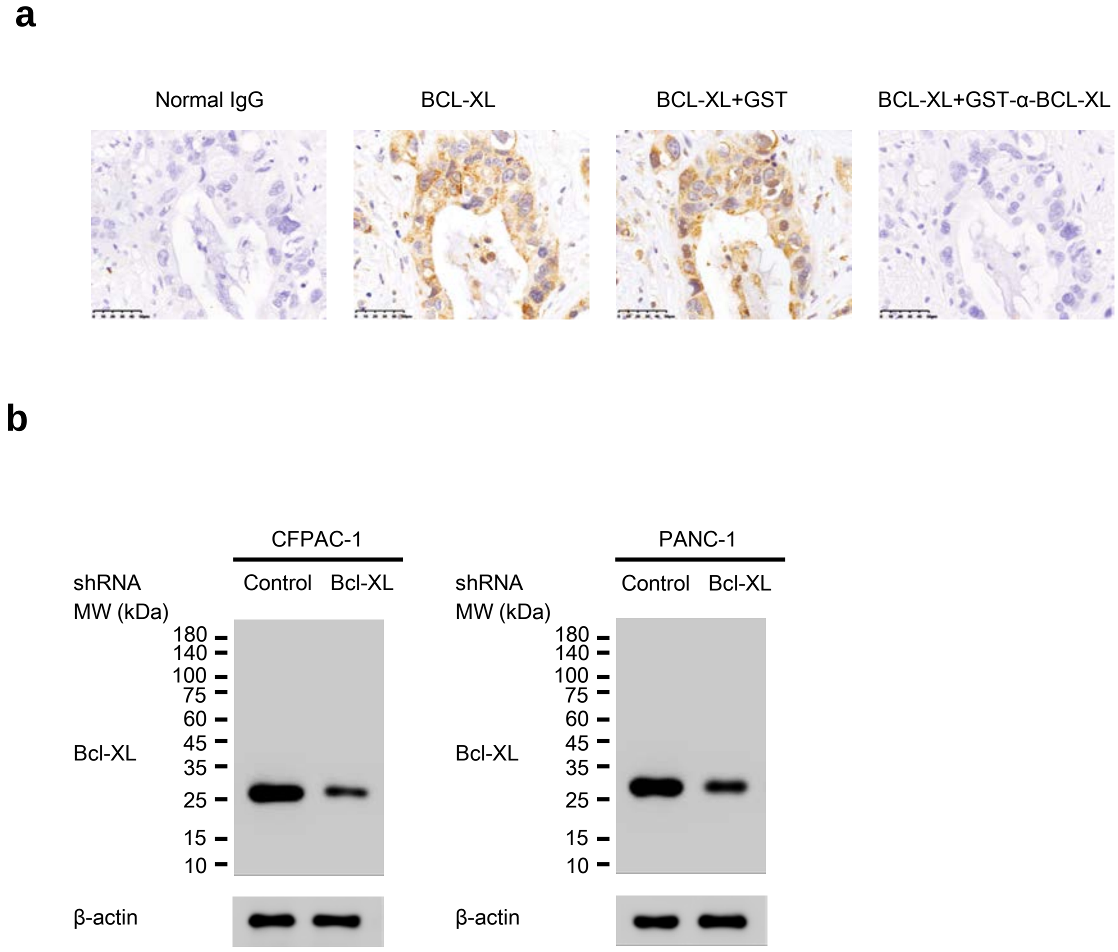

Figure S6 Validation of Bcl-XL antibody specificity

a Immunohistochemical staining of human PDAC samples incubated with normal IgG or anti- Bcl-XL. For validation of antibody specificity, The anti- Bcl-XL was incubated with recombinant GST- Bcl-XL or GST tag for 1 h before applying to tissue sections. Scale bar: 50 µm. **b** Detection of Bcl-XL in PANC-1 and PANC-1 cell lysates stably infected with control shRNA or Bcl-XL shRNA by Western blot analysis with Bcl-XL antibody.
