# Supplementary material for: GATA1 Promotes Gemcitabine Resistance in Pancreatic Cancer through Antiapoptotic Pathway
Source: J Oncol. 2019 Apr 10;2019:9474273. doi: 10.1155/2019/9474273 (PMC6481023; doi:10.1155/2019/9474273)
Supplement: Supplementary Materials — Figure S1: validation of GATA1 antibody specificity; Figure S2: GATA1 overexpression promotes cell proliferation and confers gemcitabine resistance in vitro; Figure S3: screening of target genes responsible for GATA1-mediated gemcitabine resistance; Figure S4: GATA1 regulates Bcl-XL through binding to its promoter; Figure S5: GATA1 mediates gemcitabine resistance of PDAC through Bcl-XL; Figure S6: validation of Bcl-XL antibody specificity; Table S1: Cox univariate and multivariate analysis of overall survival in PDAC patients; Table S2: Cox univariate and multivariate analysis of recurrence-free survival in PDAC patients; Table S3: the relationship of GATA1 and Bcl-XL with clinical characteristics in PDAC patients; Table S4: sequences for shRNA, qRT-PCR, and ChIP primers. [file 9474273.f1.zip › Supplementary Tables.doc]

**Table S1. Cox univariate and multivariate analysis of overall survival**

**in PDAC patients**

| Clinical factors | Univariate | | | Multivariate | | |
| --- | --- | --- | --- | --- | --- | --- |
|  | *P* | HR | 95%CI | *P* | HR | 95%CI |
| Sex(male vs female) | 0.793 | 1.050 | 0.729-1.511 | 0.312 | 0.822 | 0.561-1.203 |
| Age(＞60 vs ≤60) | 0.919 | 1.019 | 0.712-1.457 | 0.812 | 1.046 | 0.724-1.511 |
| Grade(Ⅰ,Ⅱvs Ⅲ) | 0.016 | 2.144 | 1.152-3.991 | 0.018 | 2.191 | 1.143-4.199 |
| Tumor location(Proximal vs Distal) | 0.839 | 1.039 | 0.717-1.507 | 0.519 | 1.142 | 0.763-1.708 |
| Tumor size(＞4cm vs ≤4) | 0.909 | 0.979 | 0.678-1.413 | 0.806 | 0.952 | 0.642-1.411 |
| Nodal metastasis(positive vs negative) | 0.001 | 1.862 | 1.287-2.695 | 0.001 | 1.885 | 1.279-2.779 |
| Perineuronal invasion(positive vs negative) | 0.457 | 1.146 | 0.800-1.644 | 0.571 | 1.812 | 0.609-1.314 |
| Vessels invasion(positive vs negative) | 0.0001 | 2.272 | 1.487-3.471 | 0.009 | 0.895 | 1.157-2.839 |
| GATA1(high vs low) | 0.0001 | 2.017 | 1.401-2.905 | 0.003 | 1.846 | 1.232-2.767 |
| Bcl-XL(high vs low) | 0.001 | 1.868 | 1.295-2.694 | 0.029 | 1.573 | 1.047-2.363 |

**Table S2. Cox univariate and multivariate analysis of recurrence-free survival**

**in PDAC patients**

| Clinical factors | Univariate | | | Multivariate | | |
| --- | --- | --- | --- | --- | --- | --- |
|  | *P* | HR | 95%CI | *P* | HR | 95%CI |
| Sex(male vs female) | 0.290 | 1.211 | 0.849-1.728 | 0.832 | 0.960 | 0.660-1.397 |
| Age(＞60 vs ≤60) | 0.531 | 0.896 | 0.636-1.263 | 0.504 | 0.885 | 0.660-1.397 |
| Grade(Ⅰ,Ⅱvs Ⅲ) | 0.006 | 2.307 | 1.272-4.185 | 0.012 | 2.218 | 1.190-4.135 |
| Tumor location(Proximal vs Distal) | 0.259 | 1.224 | 0.861-1.740 | 0.191 | 1.299 | 0.877-1.924 |
| Tumor size(＞4cm vs ≤4) | 0.806 | 1.045 | 0.736-1.483 | 0.971 | 1.007 | 0.683-1.486 |
| Nodal metastasis(positive vs negative) | 0.005 | 1.669 | 1.164-2.391 | 0.008 | 1.680 | 1.148-2.459 |
| Perineuronal invasion(positive vs negative) | 0.124 | 1.312 | 0.928-1.854 | 0.893 | 1.026 | 0.707-1.489 |
| Vessels invasion(positive vs negative) | 5×10^-5^ | 2.671 | 1.754-4.069 | 0.003 | 1.952 | 1.247-3.054 |
| GATA1(high vs low) | 0.0001 | 1.985 | 1.401-2.813 | 0.006 | 1.717 | 1.166-2.528 |
| Bcl-XL(high vs low) | 0.0004 | 1.871 | 1.320-2.654 | 0.018 | 1.604 | 1.083-2.376 |

**Table S3. The relationship of GATA1 and Bcl-XL with clinical characteristics**

**in PDAC patients**

| Clinical characteristics | | Total cases | GATA1expression | | *P* value | Bcl-XL expression | | *P* value |
| --- | --- | --- | --- | --- | --- | --- | --- | --- |
|  |  |  | Low | High |  | Low | High |  |
| Sex | Female | 71 | 39 | 32 | 0.278 | 39 | 32 | 0.278 |
|  | Male | 101 | 47 | 54 |  | 47 | 54 |  |
| Age | ≤60 | 84 | 40 | 44 | 0.542 | 45 | 39 | 0.360 |
|  | ＞60 | 88 | 46 | 42 |  | 41 | 47 |  |
| Grade | Ⅰ | 65 | 26 | 39 | 0.041 | 28 | 37 | 0.157 |
|  | Ⅱ,Ⅲ | 107 | 60 | 47 |  | 58 | 49 |  |
| Tumor location | Proximal | 111 | 54 | 57 | 0.633 | 53 | 58 | 0.426 |
|  | Distal | 61 | 32 | 29 |  | 33 | 28 |  |
| Tumor size | T1-T2 | 103 | 52 | 51 | 0.876 | 51 | 52 | 0.876 |
|  | T3-T4 | 69 | 34 | 35 |  | 35 | 34 |  |
| Nodal metastasis | Negative | 115 | 61 | 54 | 0.257 | 56 | 59 | 0.627 |
|  | Positive | 57 | 25 | 32 |  | 30 | 27 |  |
| Perineuronal invasion | Negative | 97 | 46 | 51 | 0.442 | 54 | 43 | 0.091 |
|  | Positive | 75 | 40 | 35 |  | 32 | 43 |  |
| Vessels invasion | Negative | 140 | 76 | 64 | 0.019 | 72 | 68 | 0.433 |
|  | Positive | 32 | 10 | 22 |  | 14 | 18 |  |
| Recurrence | Negative | 41 | 29 | 12 | 0.002 | 28 | 13 | 0.007 |
|  | Positive | 131 | 57 | 74 |  | 58 | 73 |  |
| Survival | Alive | 52 | 36 | 16 | 0.001 | 36 | 16 | 0.001 |
|  | Dead | 120 | 50 | 70 |  | 50 | 70 |  |

**Table S4. Sequences for shRNA, qRT-PCR and ChIP primers**

**Table S4a. The cDNA target sequences of shRNA and siRNA**

| **Gene** | **Target sequence (5’-3’)** |
| --- | --- |
| GATA1-1 | TAGTGCTTATGGGGGCCCTGACTTT |
| GATA1-2 | GAAGCGCCTGATTGTCAGTAAACGG |
| Bcl-XL-1 | GCTCACTCTTCAGTCGGAAAT |
| Bcl-XL-2 | GTGGAACTCTATGGGAACAAT |

**Table S4b. Primer sequences for qRT-PCR**

| **Gene** | **Forward(5’-3’)** | **Reverse(5’-3’)** |
| --- | --- | --- |
| GATA1 | GGAGACTTTGAAGACAGAG | GGAGAGGAATAGGCTGCTG |
| Bcl-XL | ATCAATGGCAACCCATCCTG | GTAAGTGGCCATCCAAGCTG |
| Survivin | AAGAACTGCCCTTCTTGGA | CAACCGGACGAATGCTTTT |
| Cyclin D2 | ACCTTCCGCAGTGCTCCTA | CCCAGCCAAGAAACGGTCC |
| CDK6 | CCAGATGGCTCTAACCTCAGT | AACTTCCACGAAAAAGAGGCTT |
| p27KIP1 | TAATTGGGGCTCCGGCTAACT | TGCAGGTCGCTTCCTTATTCC |
| c-myc | GTCAAGAGGCGAACACACAAC | TTGGACGGACAGGATGTATGC |
| p53 | GAGGTTGGCTCTGACTGTACC | TCCGTCCCAGTAGATTACCAC |
| Bcl-2 | ATTGTGGCCTTCTTTGAGTTCG | CATCCCAGCCTCCGTTATCC |
| Mcl-1 | TGCTTCGGAAACTGGACATCA | TAGCCACAAAGGCACCAAAAG |
| MDR-1 | GGGATGGTCAGTGTTGATGGA | GCTATCGTGGTGGCAAACAATA |
| NF-κb p65 | GAATGGCTCGTCTGTAGTG | TGGTATCTGTGCTCCTCTC |
| dCK | CCATCGAAGGGAACATCGCT | GGTAAAAGACCATCGTTCAGGT |
| RRM1 | ACTTCGGCTTTAAGACGCTAGA | GCATGAGTAAACCACCTCTCAGA |
| hENT1 | GTGCCTTCGGCTACTTTATCAC | GCTAATGAGGTCCAACTTGGTCT |
| ABCG2 | ACGAACGGATTAACAGGGTCA | CTCCAGACACACCACGGAT |
| Akt | TCCTCCTCAAGAATGATGGCA | GTGCGTTCGATGACAGTGGT |

**Table S4c. Primer sequences for ChIP**

| **Bcl-XL promoter** | **Forward(5’-3’)** | **Reverse(5’-3’)** |
| --- | --- | --- |
| -1029~-943 | GACTGACTGACTGACTGAATG | CTCGCGGTAGATCTGGGGAAT |
| -938~-833 | GCGATCTCTCCCCGGTCCTGAT | ACTTGCAAGCTCAGTCACTTC |
| -760~-627 | AAGAGCTCTTGCGTCTGGAAG | GCAGCCAATCAGCGAGCCCAG |
